# An unusual pigmented tonsil neoplasm presenting to Head & Neck Clinic: a case report and literature review of primary tonsillar malignant mucosal melanoma

**DOI:** 10.1093/jscr/rjac022

**Published:** 2022-02-09

**Authors:** Pete J Rae, James E H M Bates, Lisa R Fraser

**Affiliations:** 1 Oxford Medical School, Medical Sciences Division, University of Oxford, Oxford, UK; 2 Department of Otolaryngology - Head and Neck Surgery, John Radcliffe Hospital, Headington, Oxford, UK

## Abstract

Primary mucosal melanoma of the tonsil is rare, with 27 reported cases. Careful diagnosis is necessary, as the tonsil is more often a site of metastatic melanoma from a cutaneous primary tumour. In this report, we present a case of primary right tonsillar mucosal melanoma with widespread metastasis in a 31-year-old man who presented with a 3-month history of enlarging neck lumps. On examination, he had cervical lymphadenopathy and a pigmented, vascular lesion of his right tonsil, which was diagnosed as melanoma following investigation. He had widespread metastases upon presentation, and is currently undergoing palliative immunotherapy. Owing to the aggressive behaviour, late presentations and lack of effective treatment to cure mucosal melanomas, they have a poor prognosis. Treatment involves wide local excision in local disease, and immunotherapy as the first-line for metastatic disease.

## INTRODUCTION

Although melanoma is the fifth most commonly diagnosed cancer in the UK, fewer than 2% of these are mucosal melanomas [1]. Mucosal melanomas can arise from any mucosal membrane, but 55% occur in the head and neck region [2]. The tonsil, however, is a rare site for a primary mucosal melanoma, with only 27 cases having been reported in the literature [3]. This report presents a case of a primary mucosal melanoma of the right palatine tonsil.

## CASE REPORT

**Figure 1:**
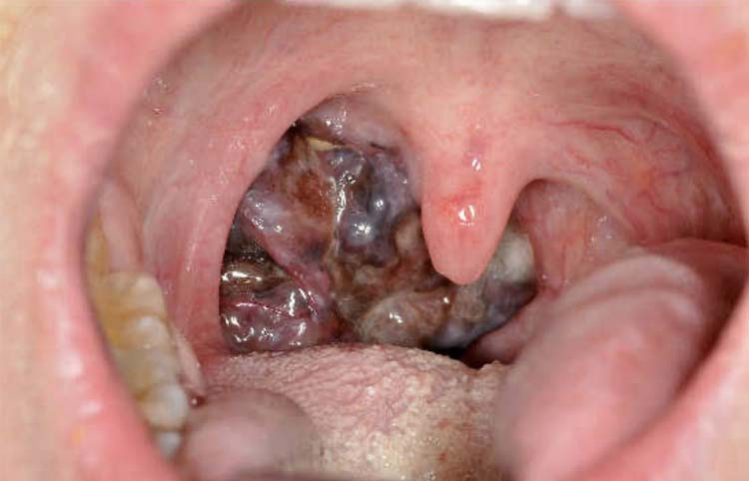
Mucosal melanoma of the right palatine tonsil.

Our patient was referred to Head and Neck Clinic with 3 months of slowly enlarging neck lumps and an abnormal right tonsil, in the absence of any other symptoms. Neck examination revealed cervical lymphadenopathy. The tonsil was enlarged and vascular (Fig. 1). The rest of the physical examination was normal. Initial blood tests revealed anaemia and thrombocytosis.

The patient was generally fit and well, with an ECOG performance status score of 0 at first presentation. He had no significant past medical, surgical or family history. His only regular medication was topical Minoxidil. He had never smoked, and reported drinking <15 units of alcohol per week.

As the patient’s presentation was suspicious for malignancy, a computed tomography (CT) neck/thorax/abdomen/pelvis with contrast was performed. This showed a right oropharyngeal mass measuring 28 × 24 ×27 mm, with nodal disease in the right cervical, anterior mediastinal, right hilar and abdominal lymph nodes. There was extranodal disease in the spleen, liver, lungs, left adrenal gland, right kidney, right pararenal space and omentum. At initial presentation, there was clinical suspicion that the patient had a high-grade lymphoma, Stage IVa.

The patient underwent an ultrasound-guided core biopsy of the largest cervical lymph node, right Levels II–IV. Histological analysis confirmed a diagnosis of metastatic melanoma originating from the right palatine tonsil. Genetic analysis showed a BRAF mutation, with no other mutations in the genes screened using the Ion Ampliseq Cancer Hotspot Panel (v2).

The patient started a course of ipilimumab and nivolumab combination immunotherapy. He was admitted 3 days after his first dose with abdominal pain and vomiting. A CT scan showed small intestine intussusception, which was managed conservatively with analgesia and co-amoxiclav. The patient’s pain resolved, and he was discharged after 3 days.

Following this, the patient returned for an magnetic resonance imaging (MRI) head with gadolinium contrast to complete his staging. The MRI scan revealed a large right cerebellar metastasis, with a 46 mm maximum diameter, causing effacement of the fourth ventricle and hydrocephalus. Alongside this, there were smaller metastatic lesions in both cerebellar hemispheres, as well as in the frontal and parietal lobes. He was referred immediately to the oncologist on-call. The patient had had new-onset vomiting, 1–2 times a day for the week before the MRI head. A neurological exam revealed right intention tremor, right dysdiachokinesia, left nystagmus and ataxic gait, in keeping with right cerebellar dysfunction due to metastasis. The patient was admitted under neurosurgery, who decided against surgical resection of the cerebellar mass and ventriculoperitoneal shunting at this stage. His ECOG performance status was updated to 1. He was started dexamethasone and a proton pump inhibitor to reduce intracranial swelling.

In light of the cerebellar metastases, the patient’s treatment was changed to encorafenib and binimetinib (a combination therapy targeting metastatic melanoma with BRAF mutations), with a view to intracranial tumour shrinkage.

## DISCUSSION

The pathogenesis of mucosal melanoma is unknown, and no risk factors have been identified. They occur equally in men and women, and the median age at diagnosis is 72 years old [4]. The most commonly mutated genes in mucosal melanomas are NRAS, BRAF, NF1 and c-KIT [5]. This is a different pattern of mutations than those seen in cutaneous melanoma. For example, BRAF is mutated in the majority of cutaneous melanomas, but only 5% of mucosal melanomas [6].

Symptoms associated with tonsillar melanomas include dysphagia, odynophagia and bleeding. Presentation with cervical lymphadenopathy secondary to metastasis to regional nodes, as in our patient’s case, is also common [3]. Around 19% of patients with head and neck mucosal melanoma present with regional lymph node metastases, and 10% with distant metastases [7]. The most common sites of metastasis are the lungs, liver, bone and brain [3].

Although 5-year survival rates for cutaneous melanomas are among the highest for any malignancy (90%), mucosal melanomas are more aggressive, often diagnosed at a later stage due to a lack of symptoms in the early stages of disease and are less treatable. As such, overall 5-year survival for patients diagnosed with mucosal melanoma is <25% [8]. Important prognostic factors for primary mucosal melanomas of the head and neck include stage, tumour thickness, surgical margin status, vascular invasion and age above 70 years [1]. Staging of mucosal melanomas of the head and neck has proven challenging—the American Joint Committee on Cancer (AJCC’s) mmTNM system has been proposed specifically for these malignancies, and has been endorsed by the British Association of Head and Neck Oncologists (BAHNO; [9]). Under this staging system, all tumours are T3 or T4, reflecting their aggressive nature. With regards to tonsillar melanomas, presentation with distant metastases indicated lower overall 5-year survival than presentation with local disease [3]. Our patient’s tumour was pigmented, as are >70% of oral melanomas [10]. Pigmented lesions are associated with tumours diagnosed at later stages, hence indicate worse prognoses than hypopigmented tumours.

The principles of management of tonsillar melanoma are assumed to be the same as those for head and neck melanoma [3]. There is still no clear consensus even with these patients, but there are recently published national guidelines, which state that all patients without distant metastases should undergo dissection of the primary tumour with clear excision margins [10]. Of these, certain patient groups should undergo sentinel lymph node biopsy with/without adjuvant radiotherapy and immunotherapy. For patients who present with metastatic disease, combination immunotherapy is the first-line treatment, with chemotherapy suggested as second-line.
